# Gut microbiota is a potential goalkeeper of dyslipidemia

**DOI:** 10.3389/fendo.2022.950826

**Published:** 2022-09-13

**Authors:** Lirong Lei, Ning Zhao, Lei Zhang, Jiamei Chen, Xiaomin Liu, Shenghua Piao

**Affiliations:** ^1^ Institute of Chinese Medicine, Guangdong Pharmaceutical University, Guangzhou, China; ^2^ Guangdong Metabolic Diseases Research Center of Integrated Chinese and Western Medicine, Guangdong Pharmaceutical University, Guangzhou, China; ^3^ Key Laboratory of Glucolipid Metabolic Disorder, Ministry of Education of China, Guangzhou, China

**Keywords:** dyslipidemia, gut microbiota, short chain fatty acid, bile acids, trimethylamine N-oxide

## Abstract

Dyslipidemia, as a common metabolic disease, could cause atherosclerosis, coronary heart disease, stroke and other cardio-cerebrovascular diseases. It is mainly caused by the interaction of genetic and environmental factors and its incidence has increased for several years. A large number of studies have shown that gut microbiota disorder is related to the development of dyslipidemia closely. Especially its metabolites such as short-chain fatty acids, bile acids and trimethylamine N-oxide affect dyslipidemia by regulating cholesterol balance. In this paper, we systematically reviewed the literature and used knowledge graphs to analyze the research trends and characteristics of dyslipidemia mediated by gut microbiota, revealing that the interaction between diet and gut microbiota leads to dyslipidemia as one of the main factors. In addition, starting from the destruction of the dynamic balance between gut microbiota and host caused by dyslipidemia, we systematically summarize the molecular mechanism of gut microbiota regulating dyslipidemia and provide a theoretical basis for the treatment of dyslipidemia by targeting the gut microbiota.

## Introduction

Dyslipidemia is a kind of metabolic disease. Its pathogenesis is complex. At present, dyslipidemia caused by high-calorie diet and lack of exercise has developed into a global public health problem, which seriously threatens human life and health. In addition, its high economic and social burden is still growing, and therefore, clinical research is focusing on understanding the pathogenesis of dyslipidemia. Gut microbiota refers to all microorganisms in the huge microecological environment in the intestine, including bacteria, fungi, viruses and so on. Studies have shown that gut microbiota plays a very important role in human nutritional metabolism, growth and development, immunity and disease occurrence. It helps to regulate material metabolism, promote vitamin synthesis, defend against pathogens, stimulate the maturation of host immune organs, activate the immune system and maintain intestinal barrier function (1, 2).

Gut microbiota and the host depend on and restrict each other, and their dynamic equilibrium is an important sign of physical health. Indeed, gut microbiota seems to exert multiple functional properties that affect human physiology and pathology (3). In recent years, the emergence of genomics, proteomics, metabolomics and other technologies has greatly promoted the study of gut microbiota-related diseases, such as intestinal diseases, neurological diseases and metabolic diseases. A recent study found that the change in gut microbiota structure is closely related to the occurrence and development of dyslipidemia (4). High-fat diet or dyslipidemia can change the intestinal environment on which gut microbiota depends, affect the reproduction and metabolism of the normal flora of the gut, leading to the imbalance of gut microbiota, and then aggravate lipid metabolism disorders and form a vicious circle (5). However, the molecular regulation mechanism of gut microbiota affecting dyslipidemia remains unclear. Therefore, this paper focuses on reviewing the relationship between gut microbiota and dyslipidemia, and analyzes the molecular mechanism of gut microbiota regulating dyslipidemia, to provide new ideas for the prevention and treatment of dyslipidemia by targeting the gut microbiota.

## The study overview on the gut microbiota and dyslipidemia

**Figure 1:**
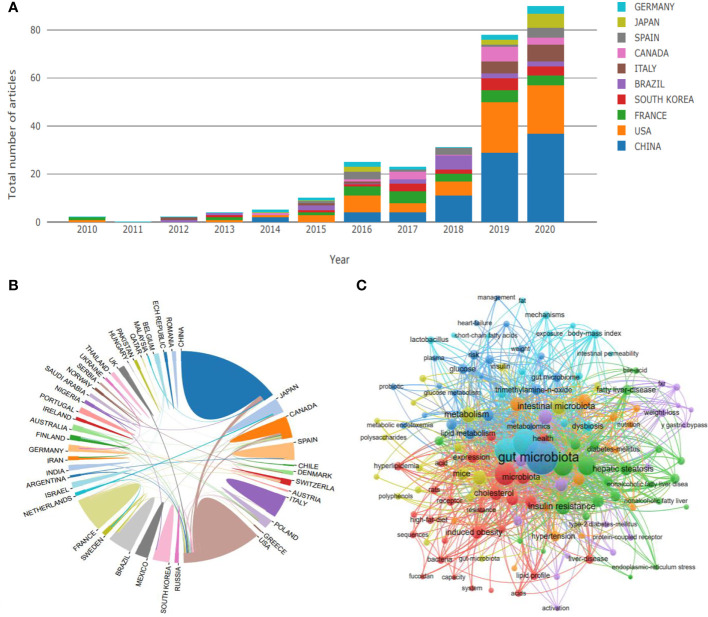
Research trend of the gut microbiota regulating dyslipidemia. **(A)** The number of published papers over the years includes the distribution of countries, **(B)** State relations, **(C)** Related keywords.

To clarify the general situation of the research on gut microbiota and dyslipidemia, we used the core set of Web of Science as the retrieval database, Dyslipidemia and Gut microbiota as the retrieval keywords, and the relevant studies on gut microbiota and dyslipidemia at home and abroad from 1990 to 2020 were retrieved. Finally, 309 pieces of literature were retrieved. After excluding irrelevant literature, 288 pieces of literature were included for the construction of the literature knowledge map. Vos Viewer software was used to conduct visual analysis with countries and keywords as research objects. The results showed that since 2012, the research on gut microbiota and dyslipidemia has attracted more and more attention, and the number of documents has increased year by year. Especially since 2019, the related literature has shown a cliff-like growth. The research on gut microbiota and dyslipidemia are dominated by China, followed by the United States, indicating that China currently has the largest research influence in this field and is in an internationally leading position in this research field (**Figure 1A**). Overall, the research on gut microbiota and dyslipidemia can be roughly divided into three stages. The first stage from 2010 to 2015 was the initial stage of research in this field, and the number of academic papers published fluctuated a little. The second stage was from 2016 to 2018. After the sudden increase in the number of literature published in 2016, the field entered a stage of slow development. The third stage was the stage of rapid development, from 2019 to 2020, the number of literature increased in a cliff-like manner, indicating that this research field has received extensive attention from scholars (**Figure 1A**). From the perspective of international research scholars and institutional cooperation, the United States is China’s most important object of international cooperation. In addition, the overall cooperation network is relatively sparse, only some institutions cooperate closely, and some marginal institutions still fail to participate in academic exchanges effectively. This reflects that the necessary academic exchanges between regions are insufficient, which limits the development of this research field to a certain extent (**Figure 1B**). In terms of keywords, much research on dyslipidemia focused on gut microbiota. In addition, keywords such as short-chain fatty acids (SCFAs), bile acids (BAs), and trimethylamine-N-oxide (TMAO) frequently appeared and showed a strong interactive relationship, suggesting that dyslipidemia was closely related to gut microbiota metabolites (**Figure 1C**).

Previously, because most microorganisms in the intestine were anaerobic, only 10%-25% of the microbiota could be isolated by conventional culture technology. With the development of anaerobic culture technology, dominant genera were identified such as Bacteroides, Clostridium, Bifidobacterium, etc. However, initial studies on gut microbial composition and function were limited by the difficulty to culture all intestinal microbes (6).

With the emergence of high-throughput gene sequencing technology, the current research on gut microbiota is mainly based on bacterial 16S rRNA sequencing technology. Sequencing of bacterial genes involves metagenomic analysis of DNA that codes for 16S rRNA. Common regions for bacterial identification in 16S rRNA are V3, V4, V6 and V8 (7). Although 16S rRNA sequencing can provide massive data with high accuracy, library preparation methods and primer selection may lead to sequencing errors (6, 8). In addition, the use of metabolomics to evaluate small molecules associated with the metabolic interrelationships of host bacteria metabolism is becoming another rapidly developing field to study gut microbiota and health and disease status.

## The interaction between diet and gut microbiota is an important factor leading to dyslipidemia

**Figure 2:**
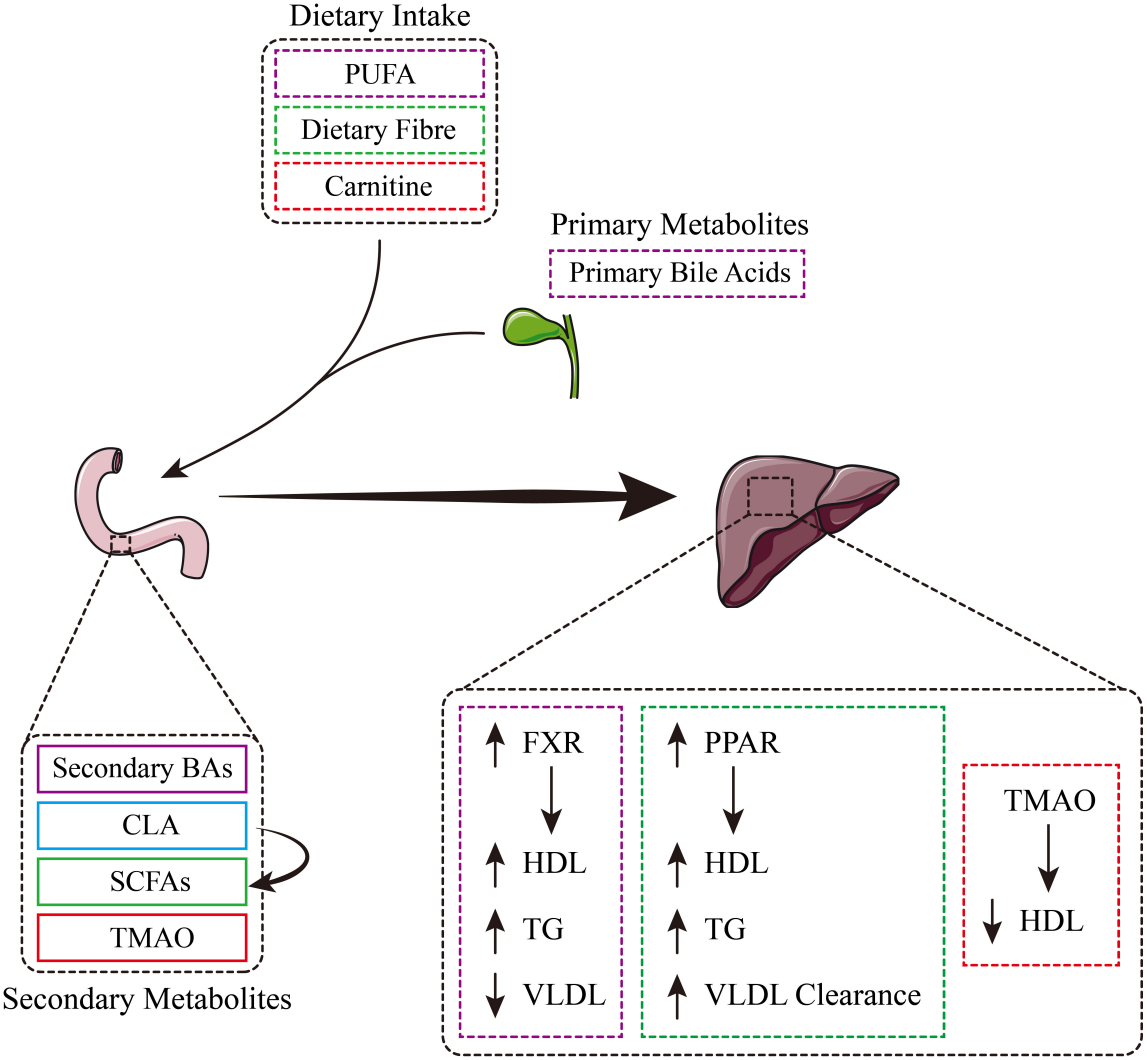
Schematic diagram of the interaction between diet, gut microbiota and lipid metabolism.

As a metabolic “organ”, gut microbiota plays a key role in host metabolism, internal balance and health maintenance, and is closely related to the occurrence and development of dyslipidemia (9). When the intestinal environment changes, the growth of normal flora such as Bifidobacterium, Lactobacillus and butyric acid-producing bacteria is inhibited, and Enterobacteria increase and dysregulation, resulting in dyslipidemia. In turn, dyslipidemia further aggravates the dysbiosis of gut microbiota. A large number of nutrients, including polyunsaturated fat (PUFA), dietary fiber, carnitine and bile acids ingested by the body, are decomposed by microbial enzymes during digestion to produce short-chain fatty acids (SCFAs), conjugated linoleic acid (CLA), trimethylamine nitrogen oxide (TMAO) and secondary bile acids (BAs) (9). The production of CLA can promote the generation of SCFAs and indirectly affect the overall yield of SCFAs (10, 11). Secondary metabolites produced in the gut play a role in different parts of the body. For example, in the liver, TMAO reduces HDL, thereby disrupting lipid metabolic homeostasis (**Figure 2**) (12). SCFAs and CLA interact with peroxisome proliferator-activated receptors (PPARs) to scavenge high levels of HDL, triglycerides (TG) and VLDL (13). Additionally, secondary bile acids’ interactions with FXR receptors are associated with high levels of high-density lipoprotein and lipolysis, as well as low levels of very low-density lipoprotein (14).

Overall, diet is the main factor affecting the composition of gut microbiota. Gut microbiota is sensitive to the amount and composition of food. It’s hard to separate the role of the gut microbiota from considerations of how food choices affect host metabolism.

Gut microbiota regulates dyslipidemia mainly by producing cholesterol oxidase to inhibit cholesterol synthesis and promote cholesterol degradation and transformation. Gut microbiota and its metabolites such as SCFAs and BAs, etc. could affect the efficiency of energy collection and the activation of the immune system, regulate chronic inflammation by changing the permeability of the intestinal barrier, and interfere with the reverse transport of cholesterol, resulting in metabolic disorders including dyslipidemia (15). Fu et al. clarified that Firmicutes and Bacteroides are the main phyla affect the changes of blood lipids, which have a great impact on levels of serum TG and HDL-C (16). For example, high-fat diet led to disturbance of lipid metabolism and energy metabolism in SD rats, accompanied by disturbance of nucleotide metabolism, the occurrence of inflammation, oxidative stress, and alteration of gut microbiota activity. Gut microbiota regulates multiple pathways, including host energy metabolism, intestinal epithelial permeability, intestinal peptide hormone secretion, and host inflammatory state, disrupting intestinal homeostasis, leading to abnormal BA metabolism, and altering the composition of SCFAs and production, thereby causing dyslipidemia (17, 18). Gut microbiota is influenced by genetics, lifestyle, diet, antibiotic treatment and other factors. Umair et al. Used 16S rRNA gene sequencing to show that high-fat diet can significantly increase the proportion of Firmicutes and Bacteroidetes in the colon, and high-fat meat protein can significantly reduce the relative abundance of serum bifidobacteria, but increase the level of lipopolysaccharide, cause the imbalance of endogenous cannabinoid receptor and promote adipogenesis (19). Therefore, improving dietary habits combined with specific drugs is a strategy to prevent and treat dyslipidemia and atherosclerosis (20).In conclusion, a large number of animal models and human studies have repeatedly demonstrated that gut microbiota disturbance is an important cause of dyslipidemia. For dyslipidemia caused by high-fat diet, the regulation of gut microbiota may be a new idea.

**Table 1** summarizes the relationship between lipid-related traits and the gut microbiota of rats, mice and other animals (**Table 1**). Studies have shown that dyslipidemia is significantly correlated with Firmicutes, Bacteroides, Haemophilus and other bacteria. In particular, Haemophilus was significantly negatively correlated with related indicators of dyslipidemia (25), suggesting that increasing Haemophilus may help reduce dyslipidemia.

**Table 1:** Examples of recent association studies between lipid-related traits and the gut microbiota in murine models.

Subjects	Dietary Supplementation	Main Findings	Reference
12-14w C57BL/6J mice	Lard/Fish Oil for 11 weeks (High-Fat Diet)	TC↑, TG↑ in lard diet	(21)
12w GF mice and CONV-R mice	High-Fat Diet for 12 weeks	CONV-R mice TC↑, LDL↑, adipose and hepatic TG↑, serum TG↓	(4)
8w male inbred SD rats	High-Fat Diet for 8 weeks	TC↑, LDL↑ in high-fat diet	(22)
2w SD Rats	High-Fat Diet + Probiotic Supplementation for 5 weeks	HDL↑, LDL↓, TC↓, TG↓ after Probiotic Supplementation	(23)
6w male golden hamsters	High-Fat Diet + RS Supplementation for 4 weeks	HDL↑, LDL↓, TC↓, TG↓ after RS Supplementation	(24)

Although there has been extensive evidence that changes in the gut microbiota are involved in the pharmacological effects of many natural medicines such as cranberry (26), Iris fruit (27). However, except for simple association, there is little evidence of a causal relationship between gut microbiota and the efficacy of drug therapy. At present, researchers have proposed several strategies to modulate the gut microbiota through prebiotics or probiotics, arguing that the amount and type of prebiotics entering the large intestine affect the growth of the microbial population (28, 29). For example, the usage of resistant starch (RS) from purple yam could ameliorate lipid metabolism and this effect is related to gut microbiota modulation (24).

It is reported that there is a negative correlation between TG, LDL levels and gut microbiota diversity (**Table 2**) (16, 32, 33), On the other hand, HDL cholesterol was found to be positively associated with microbial richness (34). In addition, long-term (≥ 8 weeks) modulation of gut microbiota can significantly reduce the levels of TC, TG and LDL-C, and increase the level of HDL-C (35). Therefore, it is recommended to use gut microbiota to treat dyslipidemia for at least 8 weeks to achieve a better therapeutic effect. However, it is currently controversial whether the relevant products of the gut microbiota help in the treatment of dyslipidemia (36).

**Table 2:** Examples of recent association studies between lipid-related traits and gut microbiome in humans.

Subjects	Dietary Supplementation	Main Findings	Reference
127 dyslipidemia subjects	Probiotic	TC↓, LDL↓ after Probiotic Supplementation	(30)
893 healthy individuals from LifeLines cohorts	-	TG↑ correlated to diversity↓ and abundance↓ of Bacteroidetcs and Proteobacteria; HDL↑ correlated to diversity↑	(16)
34 moderately dyslipidemia subjects	Probiotic	TC↓ after Probiotic Supplementation	(31)

## Gut microbiota mainly affects the development of dyslipidemia in three pathways

**Figure 3:**
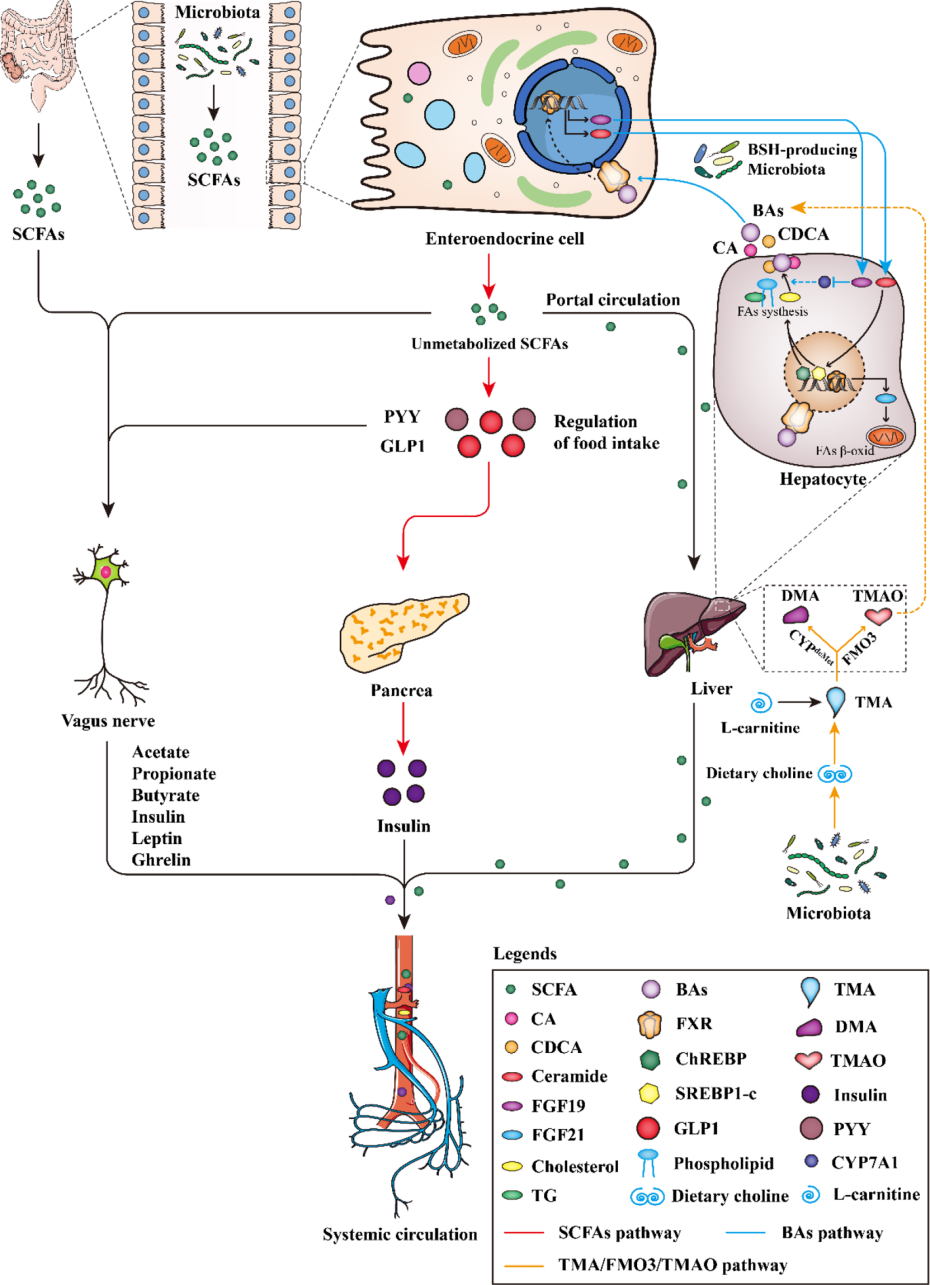
The gut microbiota regulates the pathway of dyslipidemia.

Firstly, short-chain fatty acids (SCFAs), the main metabolites of gut microbiota, could reduce the activity of liver lipid metabolism enzymes and redistribute blood cholesterol to the liver, to regulate lipids metabolism. Secondly, gut microbiota can produce bile salt hydrolase, which can affect cholesterol metabolism by regulating the intestinal hepatic circulation of bile acids. Thirdly, gut microbiota mainly affects lipid metabolism, cholesterol transport (RCT) and bile acids metabolism by regulating TMA/FMO3/TMAO pathway (**Figure 3**).

### Short-chain fatty acids pathway

SCFAs can reduce the activity of liver lipid metabolism enzymes and regulate the distribution of cholesterol in blood and liver, to reduce the levels of serum triacylglycerol and cholesterol. SCFAs can also strengthen the protective effect of the intestinal barrier, defend intestinal antigens, affect the permeability of intestinal epithelial cells, increase the sense of satiety and participate in the change of flora microenvironment (37, 38).

The increase or decrease in the number of SCFAs can improve or aggravate the occurrence and development of dyslipidemia. (**Figure 3** red line). Once produced, SCFAs are readily absorbed and catabolized by intestinal epithelial cells. Most acetate bypasses splanchnic circulation, is oxidized by muscle or used for lipogenesis by adipocytes (39). Perry et al. found that acetate, when produced in elevated quantities by the gut microbiota, could activate the parasympathetic nervous system, promoting glucose-stimulated insulin secretion, increasing the appetite, and eventually leading to imbalanced lipid metabolism (40). Propionate stimulates the release of the satiety hormone peptide YY (PYY) and glucagon-like peptide 1 (GLP-1) in the intestine, thereby reducing energy intake (**Figure 3**). PYY and GLP1 stimulate the secretion of insulin by the pancreas. Insulin enters the blood circulation, promotes the conversion of glucose into fat, stimulates fat production, inhibits fat decomposition, and further leads to dyslipidemia (**Figure 3**). Acetate and propionate took up by the liver were used as substrates for lipogenesis and gluconeogenesis, which increased energy dissipation. In addition, propionate can also indirectly induce intestinal gluconeogenesis (IGN) through brain-enteric nerve connections. In conclusion, the increase of propionate may increase the risk of dyslipidemia (41). Butyrate can further activate macrophages by activating the GPR109A receptor and reduce the activity of total lipase to inhibit the synthesis and accumulation of triglycerides and regulate blood lipid homeostasis, its increase can inhibit the growth of body weight in obese mice induced by high-fat diet (42). In addition, SCFAs can also directly activate vagal afferent and affect the number and function of islets β cells, promote liver gluconeogenesis metabolism and provide 30% energy for liver metabolism (37, 38) (**Figure 3**). Importantly, butyrate probably acted directly on vagal afferent terminals, it can promote the growth, migration and differentiation of intestinal epithelial cells, provide essential nutrients for intestinal epithelial cells and muscles, improve the energy metabolism of intestinal epithelial cells, maintain the stability and integrity of intestinal mucosa, and prevent the invasion of pathogenic bacteria and lipopolysaccharide (LPS), and stimulate the secretion of GLP-1 by L-type cells in the intestinal mucosa (41, 43).

Supplementation of fermentable fiber bitter melon in HFD-fed mice prevents dyslipidemia by promoting bacterial production of SCFAs and restoring gut microbiota composition (44). In addition, increasing the abundance of SCFAs producing bacteria or supplementing SCFAs has the significant lipid-lowering effect (45, 46). The latest study shows that Bupleuri radix extract can improve lipid metabolism in high-fat diet-induced obese mice by modulating the FGF21 signaling pathway mediated by gut microbiota (47). These data highlight that SCFAs are bioactive by-products that interact with host metabolism in complex ways. However, whether the end effect is positive or negative may be highly context-dependent. Interestingly, SCFAs may exert their beneficial effects in part through specific G protein-coupled receptors, so activation of G protein-coupled receptors with specific agonists is a prospective strategy for the treatment of dyslipidemia.

### Bile acids pathway

Bile acids, as signal molecules, bind to bile acid receptors and exert various biological effects *in vivo*. Gut microbiota interferes with cholesterol metabolism by regulating bile acid enterohepatic circulation, thereby affecting dyslipidemia (**Figure 3** blue line). Le Roy et al. used broad-spectrum antibiotics to deplete the gut microbiota of hypercholesterolemic female Apoe^-/-^ mice and measured plasma cholesterol levels as well as cholesterol synthesis and flux by complementary methods, showing that gut microbiota strongly regulates plasma Cholesterol levels, hepatic cholesterol synthesis, and enterohepatic circulation, demonstrating that specific gut microbiota composition modulates cholesterol absorption, biosynthesis, and circulating cholesterol levels (48).

BAs have lipid-digesting functions and are novel metabolic regulators (49). Regulation of gut microbiota and BAs metabolism is a key target for the treatment of dyslipidemia, and the regulation of microbiota can cause changes in BAs composition, which in turn affects the treatment of metabolic diseases. For high-fat diet-induced gut microbiota disturbances, BAs can destroy gut bacteria, and gut microbiota, due to their defense mechanisms against bile acid toxicity, make cholesterol and BAs undergo a series of chemical modifications that affect their signaling mechanism (50). Indeed, increased levels of primary bile acid CDCA lead to very low-density lipoprotein production and decreased plasma triglyceride concentrations. Primary bile acids such as cholic acid (CA) and chenodeoxycholic acid (CDCA) can promote the growth of Clostridium difficile pathogens. Secondary bile acids such as deoxycholic acid and lithocholic acid can inhibit the proliferation of Clostridium difficile. Primary BAs are converted into secondary BAs by microbe-associated bile-salt hydrolase (BSH) and 7-dehydroxylase under the influence of gut microbiota in the terminal ileum and colon. When primary BAs cannot be converted to secondary BAs, it may lead to Clostridium difficile infection, resulting in severe gut inflammation (51). Gut microbiota can regulate lipid metabolism by balancing the BA pool and composition. Short-term supplementation of antibiotics to mice can reduce secondary BAs bacteria, liver deoxycholic acid (DCA) and lithocholic acid concentrations and serum triglyceride levels. This suggests that secondary BAs can act as a regulator to maintain metabolic host homeostasis (52) (**Figure 3**). In addition, TGR5 can reduce the size of the bile acid pool by enhancing the negative feedback regulation of bile acid synthesis, and stimulate intestinal endocrine cells to secrete GLP-1 (53). Bile acids can act as ligands to activate FXR and TGR5 to participate in the regulation of glucose and lipid metabolism, thereby regulating lipid metabolism and affecting blood lipid content (54). Bifidobacterium, Lactobacillus acidophilus and Bacteroidoides Families can produce bile hydrolytic enzyme (BSH), regulate bile acids enterohepatic circulation, affect FXR activity, regulate FXR metabolic pathway, and thus interfere with cholesterol metabolism. Steroid dehydrogenases produced by Firmicutes, Streptococcus, and Bacillus aerogenes can catalyze the production of secondary bile acids and convert bound bile acids into secondary free bile acids. Secondary free bile acids can regulate lipid metabolism in the liver and whole body through G-protein-coupled receptors. The imbalance of bacterial flora can lead to a disorder of bile acids secretion, resulting in dyslipidemia (14, 55–57). In addition, cholesterol 7α-hydroxylase (CYP7A1) is a rate-limiting enzyme for bile acid synthesis. A variety of nuclear receptors participate in the regulation of CYP7A1 gene binding sites, which can promote its activity and increase bile acids synthesis, and play a key role in maintaining cholesterol metabolism balance *in vivo (* 58). FXR is highly expressed in the liver and ileum. FXR expressed in the liver regulates CYP7A1 by inducing the expression of small heterodimers (SHP). SHP can bind to liver receptor homolog-1 (LRH-1), thereby inhibiting CYP7A1 expression. FXR expressed in the ileum regulates CYP7A1 through dependencies on a fibroblast growth factor (FGF19) mechanism (53, 59) (**Figure 3**). BAs can inhibit intestinal FXR signaling, increase fibroblast growth factor FGF19 production, ultimately promoting BA synthesis and decrease cholesterol levels. FXR is also a nuclear receptor, which regulates the protein expression of bile acids uptake and transport in the intestine and its circulation. The BAs flow out from the gallbladder and controls the absorption of bile acids in the intestine and the liver (60).

Dyslipidemia and steatosis in the liver can communicate and interact with gut microbiota through BAs (61). Given the potential benefit of BAs with synthetic agonists of FXR and FGR5 in dyslipidemia (62), Researchers have found effective agonists for the synthesis of FXR and TGR5 and are currently in phase II or phase III clinical trials (63, 64). The steady-state response of bile acid manipulation to homeostasis is a huge challenge, which means that the overall impact on the size and composition of the BAs pool reaching the target tissue is usually difficult to predict (65).

### TMA/FMO3/TMAO pathway

Gut microbiota intervenes lipid metabolism by regulating the TMA/FMO3/TMAO pathway, affecting reverse cholesterol transcription (RCT) and BAs metabolism, regulating cholesterol level and affect blood lipid (66, 67) (**Figure 3**, orange line). Studies have shown that the expression or activity of liver FMO3 is sensitive to sex steroids, dietary factors, calorie intake, no signal transduction and transcription regulator CCAAT and enhancer binding protein (68, 69). Furthermore, nuclear FXR involved in bile acid metabolism in the liver and small intestine has been found to regulate FMO3. FMO3 may cause dyslipidemia by regulating multiple pathways involved in hepatic lipogenesis and gluconeogenesis, while affecting intestinal cholesterol output and macrophage-specific RCT, disrupting cholesterol homeostasis (70, 71). Studies suggest an obligate role for the gut microbiota in converting dietary phosphatidylcholine to the pro-atherosclerotic molecule TMAO (66, 72). In this context, Choline, which contains a part of trimethylammonium, is directly converted into TMA by gut bacteria (73), then TMAO is metabolized into the blood through liver FMO3. TMAO can up-regulate macrophage receptors, reduce CYP7A1 and CYP27A1 expression of some BAs transport genes in the liver, interfere with RCT and regulate cholesterol balance, thereby affecting blood lipids (**Figure 3**). Carnitine is an important component of fatty acid metabolism. It transports activated long-chain fatty acid acyl groups to the mitochondrial matrix (74). L-carnitine is produced by lysine in eukaryotes and is catabolized by prokaryotes. Prokaryotes begin to produce TMA and apple paraldehyde through the cleavage of the main chain carbon-nitrogen bond (75) (**Figure 3**). Treatment with dietary carnitine directly, resulted in a reduction of the bile acid pool in mice (76). This would have likely an effect on FXR activation. This potential interplay in the liver between TMAO and FXR is intriguing given the attention that has been paid to microbial activation of FXR. TMAO can also promote cell apoptosis, and its increase is related to the increase of Clostridium, Escherichia and Proteus (77, 78). Importantly, TMAO and butyrate, as molecules released by microbiota, may lead to dyslipidemia, impair myocardial healing or cause heart failure, and its mechanism is still speculative. High-fat diet can induce changes in the gut microbiota of mice, significantly inhibit mRNA level and protein expression of FMO3 and then reduce the concentration of TMA and TMAO, resulting in dyslipidemia (79). The increase of TMAO can promote inflammation, damage vascular function and structure, and promote dyslipidemia through inflammation, oxidative stress, up-regulation of clearance receptor (SR), RCT inhibition and cardiovascular dysfunction (80). High concentration of TMAO can also induce platelet hyperreactivity and enhance thrombus formation potential by amplifying intracellular Ca^2+^ release (81, 82). TMAO may promote cholesterol uptake by macrophages by inducing scavenging receptors CD36 and SRA1, both of which are involved in the intracellular accumulation of modified lipoproteins. Under normal dietary conditions, TMAO does not affect the plasma cholesterol level of FMO gene-deficient mice. The recognition of TMAO receptor confirms the potential association between TMAO and cholesterol level (72, 83).

## Conclusions

Gut microbiota plays a central role in the physiology and physiopathology of the development of dyslipidemia. SCFAs, BAs and TMAO are closely related to the pathogenesis of dyslipidemia. SCFAs derived from the fermentation of dietary fiber, could prove protective in the development of dyslipidemia and metabolic syndrome, while other bacterial metabolites, such as secondary BAs, TMA, maybe drivers or strong contributors. In addition, gut microbiota metabolites are also closely related to glycolipid metabolism disorders such as type 2 diabetes and non-alcoholic fatty liver disease (84, 85). Therefore, the establishment of drug development and clinical treatment targeting gut microbiota may provide technical ideas for dyslipidemia and dyslipidemia-related metabolic diseases, such as type 2 diabetes and non-alcoholic fatty liver disease. At the same time, the study of gut microbiota metabolites and their host receptors, potentially in combination with dietary interventions and lifestyle, is a promising target for the development of new therapeutic tools for chronic diseases.

## Author contributions

LL conceptualized and wrote this manuscript. NZ visualization. LZ, JC and XL revised and reviewed the format. SP assisted with the edited version and acquired the funding. All authors contributed to the article and approved the submitted version.

## Funding

This work was financially supported by the Nature Natural Science Foundation of China (81973707).

## Conflict of interest

The authors declare that the research was conducted in the absence of any commercial or financial relationships that could be construed as a potential conflict of interest.

## Publisher’s note

All claims expressed in this article are solely those of the authors and do not necessarily represent those of their affiliated organizations, or those of the publisher, the editors and the reviewers. Any product that may be evaluated in this article, or claim that may be made by its manufacturer, is not guaranteed or endorsed by the publisher.
